# Action of polystyrene nanoparticles of different sizes on lysosomal function and integrity

**DOI:** 10.1186/1743-8977-9-26

**Published:** 2012-07-12

**Authors:** Eleonore Fröhlich, Claudia Meindl, Eva Roblegg, Birgit Ebner, Markus Absenger, Thomas R Pieber

**Affiliations:** 1 Center for Medical Research, Medical University of Graz, Graz, Austria; 2 Department of Internal Medicine, Division of Endocrinology and Nuclear Medicine, Medical University of Graz, Graz, Austria; 3 Institute of Pharmaceutical Sciences, Department of Pharmaceutical Technology, Karl-Franzens-University of Graz, Graz, Austria

**Keywords:** Nanoparticles, Lysosomes, Cathepsin B, Lysosomal sulfatase, Accumulation

## Abstract

**Background:**

Data from environmental exposure to nanoparticles (NPs) suggest that chronic exposure may increase the incidence of lung, cardiovascular and neurodegenerative diseases. Impairment of cell function by intracellular accumulation of NPs is also suspected. Many types of NPs have been detected in the endosomal-lysosomal system and, upon repeated exposure, alterations of the endosomal-lysosomal system may occur. To identify such effects we compared the effect of carboxyl polystyrene particles (CPS) of different sizes (20-500 nm) on lysosomes of the endothelial cell line EAhy926 after short (24h) and long (72h-96h) exposure times. Lysosomal localization of CPS, as well as lysosomal pH, lysosomal membrane integrity, morphology of the endosomal-lysosomal system and activities of the lysosomal enzymes,cathepsin B and sulfatases, upon exposure to CPS were recorded.

**Results:**

CPS in sizes ≤100 nm showed high co-localization with lysosomes already after 4h, larger CPS after 24h. None of the particles at non-cytotoxic concentrations caused marked changes in lysosomal pH or destroyed lysosomal membrane integrity. At 24h of exposure, 20 nm CPS induced significant dilatation of the endosomal-lysosomal system and reduced activity of lysosomal sulfatases. After 72h, these alterations were less pronounced.

**Conclusions:**

Despite accumulation in lysosomes CPS induced only small changes in lysosomes. Upon longer contact, these changes are even less pronounced. The presented panel of assays may serve to identify effects on lysosomes also for other NPs.

## Background

Nano-sized materials are promising tools for new technologies in industrial, pharmaceutical and medical applications. On the other hand, nanoparticles (NPs) may cause adverse cellular events ranging from acute cytotoxicity, induction of inflammation to genotoxic effects [1]. Even if acute adverse cellular effects are not as obvious as, for instance, cell death and inhibition of proliferation, biopersistance and accumulation of NPs may interfere with the physiological function of cells and organs.

The increased incidence of several types of chronic diseases, mainly of the respiratory and cardiovascular system but also neurodegenerative diseases, after long term exposure to particulate matter is mentioned in numerous studies e.g.[2]. Also smaller effects such as impairment of cognitive function have been reported [3]. Mechanisms for the pathogenic action include generation of reactive oxygen species and induction of inflammation [4]. In addition, insufficient degradation of protein aggregates in neurodegenerative diseases (e.g. tau protein in Alzheimer’s disease, α-synuclein in Parkinson’s disease) by lysosomes induced by NPs may represent an additional causative factor.

Biopersistance of NPs has been demonstrated in several studies. After only one injection quantum dots were detectable in liver and spleen for 6 months [5,6], titanium dioxide NPs persisted for 28 days [7,8] and gold NPs were seen after 8 days [9]. When rats were exposed by inhalation to 20 nm and 250 nm TiO2 particles persistence, alteration of lung histology and of macrophage function at one year post-exposure were much more pronounced for the small particles [10]. As NPs are also taken up by non-phagocytic cells, biopersistance may also occur in these cells and lead to impaired cell function.

Long-term effects of NPs are mainly investigated by in-vivo experiments. After repeated inhalative exposure to silver and titanium dioxide NPs, minimal histopathological changes at the portal of entry and alterations in white blood counts were seen [11-13]. Other effects of NPs, like a reduced anti-bacterial defence, were only identified when animals, repeatedly exposed to diesel exhaust particles, were subjected to challenge with listeria pathogens [14].

**Figure 1:**
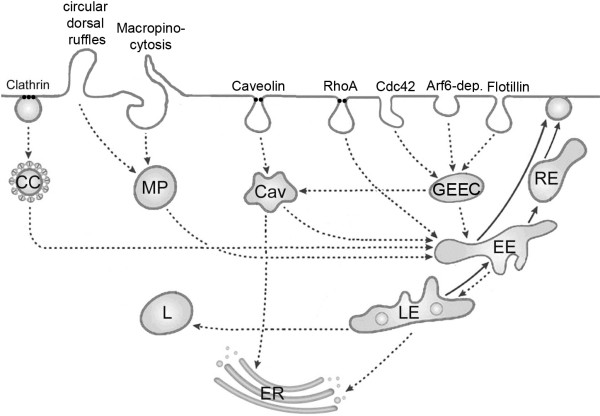
**Schematic simplified representation of potential routes for active uptake of in non-phagocytic cells.** Mechanisms of endocytosis include clathrin-dependent and clathrin-independent routes. Clathrin-dependent uptake occurs via clathrin-coated pits (CC). Clathrin-independent routes include ingestion by macropinocytosis and circular dorsal ruffles via macropinosomes (MP), caveolae-mediated endocytosis via caveosomes (Cav) and various non-clathrin non-caveolae mediated processes. The later can be subdivided into RhoA/IL2β pathway, Arf1/Cdc42, flotillin and Arf6 endocytosis. Whereas RhoA/IL2β receptor uptake involves specific tubulovesicular endosomes, the content of Arf1/Cdc42, flotillin and Arf6 endocytotic vesicles is delivered to glycophosphatidylinositol-anchored protein-enriched compartments (GEEC). Caveosomes may deliver their content to the endoplasmatic reticulum (ER) and to early endosomes (EE). The other routes transport the ingested material via endosomes (EE) and late endocytotic vesicles (LE) to lysosomes (L). Late endocytotic vesicles may also contact the endoplasmic reticulum. Recycling of membranes occurs via recycling endosomes (RE).

To minimize the influence of adaption it may be advantageous to look at organelles, where accumulation is most likely. NPs are taken up by passive and active mechanisms. Passive uptake of NPs was seen for titanium dioxide particles by adhesive interaction [15] or by passive diffusion for titanium dioxide and gold particles up to a size of 200 nm [16]. The passage of NPs through membrane protein channel has been proposed but experimental proof is lacking [17]. Active entry in non-phagocytic cells occurs via various mechanisms of endocytosis, which are enumerated without going into detail. Clathrin-mediated uptake, caveolae, macropinocytosis and clathrin- and caveolae-independent uptake were identified. Typical classifications employ coating proteins, GTPases or the absence of lipid rafts for discrimination of different clathrin- and caveolae-independent endocytotic mechanisms. According to the type of GTPase Arf6-dependent, Cdc42/Arf1-dependent and RhoA-dependent endocytosis can be discerned. Presence of the coat protein Flotillin is characteristic for Flotillin-dependent endocytosis [18]. Another nomenclature employs the term clathrin-independent carriers/glycophosphatidylinositol (GPI)-anchored protein enriched compartment (GEEC)-type endocytosis as synonym for Cdc42/Arf1-dependent endocytosis and IL-2Rβ-dependent endocytosis for RhoA-dependent endocytosis [19] (Figure 1). The material, which is taken up by endocytosis, is transported by clathrin-coated pits, macropinosomes, caveosomes, glycophosphatidylinositol (GPI)-anchored protein enriched compartments and other sorting endosomes mainly to lysosomes. The non-degradative route of caveolin-dependent uptake may also deliver the content of the caveosome to the endoplasmic reticulum and the Golgi apparatus. Spherical NPs made from iron oxide, polystyrene, gold, cadmium selenide (quantum dots) and titanium dioxide as well as nanodiamonds appear to be stored in lysosomes [20-26]. They can accumulate there because degradation of inorganic NPs in lysosomes is unlikely.

Acute damage of lysosomes may occur through oxidative stress; lysosomal membranes are especially sensitive to reactive oxygen and nitrogen species [27]. For some particles like fullerenes and quantum dots cytotoxicity by lysosome membrane damage has been reported [28,29]. For prolonged exposure to NPs organelle damage by accumulation may also occur. Change in lysosome morphology and decrease of lysosomal membrane stability by accumulation of non digested NPs has already been shown for NPs from glass wool by Koehler et al. [30]. The effects of accumulation products on lysosomes have been studied for the physiological accumulation product lipofuscin, the lipid-containing residues of lysosomal digestion. Lipofuscin in lysosomes can cause increases in lysosomal pH and inhibition of lysosomal enzymes [31]. Garnett and Kallinteri [32] suggested that accumulation of NPs might cause similar symptoms as lysosomal storage diseases. Inherited lysosomal storage diseases are good examples of the importance of lysosomal enzymes for cell function. Deficiency of specific lysosomal enzymes, for instance lysosomal sulfatases arylsulfatase A, B and G, causes lysosomal storage diseases [33]. The impaired autophagic delivery of bulk cytosolic contents to lysosomes finally resulting in accumulation of toxic protein, cellular damage and apoptosis leads to a wide spectrum of symptoms ranging from movement disorders, seizures and dementia to hepatomegaly and splenomegaly, and pulmonary and cardiac problems [34]. Drug-induced lysosomal phospholipidosis and inherited lysosomal storage disorder share analogies regarding clinical symptoms and molecular mechanisms [35]. Although it is not expected that the accumulation of NPs can cause symptoms as severe as those seen in inherited lysosomal storage diseases, such effects should be included in a realistic risk assessment of particle exposure.

For this study we used carboxyl polystyrene particles (CPS) because these particles can be obtained in reproducible quality, in a wide size range and in core-labelled fluorescent form allowing localization and tracking in living cells. In addition, they are not bio-degradable and the effect of intracellular accumulation can be studied. These particles show a size-dependent toxicity [36,37], which allows the comparison of cytotoxic and non cytotoxic particles with the same composition and under the same conditions. Intracellular distribution, lysosomal stability, pH and enzyme function after short and longer exposures were studied. To avoid dilution of the particles by cell division, cells were growth retarded by serum reduction.

## Results

### Characterization of the *in-vitro* model

#### Cell culture

Pilot experiments showed that upon cell proliferation the intracellular content of particles decreases rapidly. Therefore, cell proliferation has to be prevented when an effect of particles accumulation is studied. In order to produce viable non-proliferating cells, the serum concentration in the medium was decreased. The concentration of serum was chosen according to cell number and viability. Requirements for the exposure conditions were: variation in the cell number ≤ 15% between day1 and day5 and viability of the cells ≥ 85%. At a concentration of 2% FBS in the medium these requirements were fulfilled. At 1% FBS cell number and viability of the cells were lower and at 5% FBS cell number was too high.

To find out if serum reduction would induce changes, especially in lysosomal genes, gene-expression was analyzed. De-regulated genes were involved in primary metabolic and cellular processes, cell communication, signal transduction and response to stimuli (supplementary information, Additional file 1: Table S1 A, B). A search for de-regulation of lysosomal genes showed no hits. (The complete list of the de-regulated genes is provided as supplementary information, Additional file 2: Table S2).

#### Carboxyl polystyrene particles (CPS)

For the interpretation of the experiments potential aggregation of particles and surface charge are important as these parameters are influenced by pH, ionic strength and protein content. To this aim hydrodynamic size and surface charge of the CPS particles were determined in the medium used for testing (Table 1). Compared to the nominal size of the CPS, increases in DMEM + 2% FBS medium were more pronounced for the small 20 nm and 40 nm CPS, where 2-3.35-fold increases in size were noted compared to 1.01-1.7 fold increases in size for the larger ≥ 100 nm CPS. Surface charge ranged between -11.3 and -14.7 mV for all particles.

**Table 1:** Overview of the changes in size of the carboxyl polystyrene particles (CPS) in cell culture media (DMEM) with 2% fetal bovine albumin (FBS) and indication of surface charge (zeta potential)

**Size (nm)**	**Size (nm)**	**ζ-pot.(mV)**	**Increase in size**
**CPS (nominal size)**	**DMEM, 2% FBS**	**DMEM, 2% FBS**
20	67	−11.3	3,35
40	80	−14.7	2,00
100	170	−12.1	1,70
200	254	−12.5	1,27
500	543	−11.5	1,09
1000	1407	−11.3	1,41

#### Cellular particle uptake

The amount of cellular particle uptake was size-dependent and increased from 24h to 48h (Table 2). While the number of intracellular particle numbers was highest for 20 nm, the relative rate of ingested particles was lowest. The factor 10 in the size difference between 20 nm and 200 nm CPS resulted in about 20 times more particles, which were taken up by the cells. Normalized to the applied dose, however, only about 5% of the 20 nm CPS compared to 17% of the 200 nm ones were taken up by cells after 24h of exposure.

**Table 2:** Uptake rates of fluorescently labeled carboxyl polystyrene particles (FS) by EAhy926 cells given as number of particles (N) and percentage of applied dose (%)

	**24h**		**48h**		**72h**	
**Size (nm)**	**N**	**%**	**N**	**%**	**N**	**%**
20	8.7*10^5	4.6	9.5*10^5	8.7	9.3*10^5	8.9
40	9.6*10^4	4.9	1.2*10^5	9.1	1.3*10^5	8.6
100	1.6*10^4	10.4	2.0*10^4	20.6	2.2*10^4	22.9
200	4.7*10^3	17.2	5.3*10^3	41.9	5.3*10^3	40.9
500	3.8*10^2	28.4	5.8*10^2	67.9	5.4*10^2	70

### Cell damage and oxidative stress upon particle exposure

**Figure 2:**
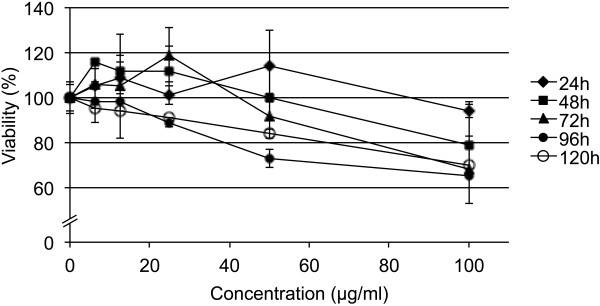
**Dose-dependent change in viability assessed by formazan bioreduction in EAhy926 cells exposed to 20 nm CPS in DMEM + 2%FBS for different times.** Decreases are related to the respective not CPS-exposed cultures as 100% at 24h-48h-72h-96h and 120h of exposure. At doses (10, 20, 40 μg/ml) and exposure times (72h) used for the studies on lysosome metabolism, no significant decrease in viability is seen (p < 0.05).

Cells exposed to CPS for different times showed a dose-dependent decrease in viability for the 20 nm CPS while no decrease of viability was seen in the case of 200 nm CPS. The dose-dependent decrease in viability was steeper when cells were exposed for 48-120h with the 20 nm CPS (Figure 2). After 120h of exposure viability had decreased to 70 ± 5% compared to 94 ± 3% after 24h. At 50 μg/ml 20 nm CPS upon 72h of exposure viability was only slightly decreased (92 ± 12%) compared to untreated cells. No decrease in cell viability at any time point was seen up to 100 μg/ml 200 nm CPS. As expected for a fluorescence core-labelled particle no differences in cytotoxicity were observed between CPS and the labeled counterparts (FluoSpheres®, data not shown). For the exposures we chose concentrations of 10, 20 and 40 μg/ml for all CPS to exclude a cytotoxic effect.

To find out if at these concentrations of CPS, oxidative stress was induced, the oxidation of the non-fluorescent dichlorodihydrofluorescein to fluorescent dichlorofluorescein was exploited. After 24h of incubation with 50 μg/ml 20 nm CPS the fluorescence increased to 135.7 ± 44% of that of the untreated controls compared to 363 ± 48% in the positive control (200 μM H_2_O_2_). Fluorescence after exposure to 50 μg/ml 200 nm CPS reached 116 ± 1.4% of the untreated controls (data not shown). The increases caused by the CPS did not reach the level of significance.

### Localization in lysosomes

**Figure 3:**
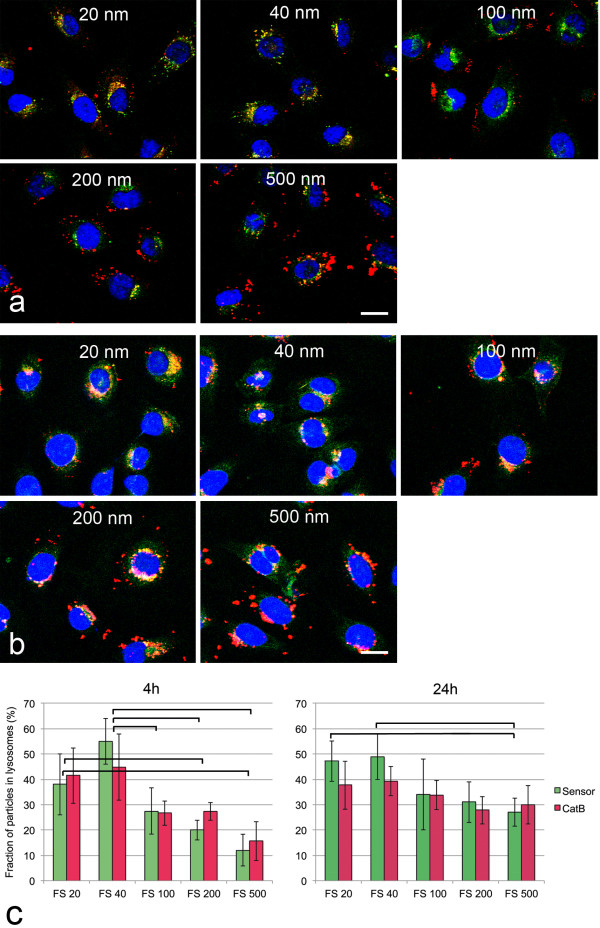
**Co-localization of fluorescently labeled CPS (FluoSpheres, 25 μg/ml in DMEM + 2%FBS) and lysosomes identified by LysoSensor and cathepsin B activity by CV-(RR)**_**2**_**substrate in EAhy926 cells after 4h of exposure (a) and 24h (b) after particles were removed from the incubation medium.** a: Co-labelling of FluoSpheres (FS red) with LysoSensor as marker for lysosomes (green). Scale bar: 10 μm. After 4h co-localization (yellow) is seen for 20 nm and 40 nm FS while 100 nm, 200 nm and 500 nm FS are seen predominantly at the cell periphery. b: After 24h more 100 nm-500 nm FS co-localize with lysosomes and only 500 nm FS were seen at the cell periphery. c: quantification of the co-localization by Metamorph® software: the pH-dependent dye LysoSensor (Sensor, green) and enzymatic activity for the lysosomal enzyme cathepsin B (CatB, red) are used as markers for lysosomes. Co-localization rates of FS with LysoSensor were slightly higher than those with CatB substrate. After 4h significant differences in the co-localization rates were seen in the combinations: 20 nm FS versus 200 nm and 500 nm FS and 40 nm FS versus 100nm, 200 nm and 500 nm FS. After 24h only the differences between 20 nm and 40 nm FS on the one hand and 500 nm FS were significant. Particles with significant differences in the co-localization rates (p < 0.05) are linked by brackets.

To find out if CPS at a single exposure of 25 μg/ml accumulate in lysosomes co-localization of fluorescent CPS (FluoSpheres®) and lysosomes identified by activity for cathepsin B and pH sensitive dye was performed. The majority of organelles stained with both LysoSensor and cathepsin B substrate but small organelles in the cellular periphery reacted only with LysoSensor (Additional file 3: Figure S3). Pictures taken immediately after removal of the particles after 4h of incubation and after 24h of post-incubation in medium showed that the 20 nm and 40 nm particles co-localized with lysosome markers whereas many 200 nm and 500 nm FS were still located at the cell periphery (Figure 3a, co-localization with LysoSensor shown). After 24h particles of all sizes were seen inside the cells and only differences in the co-localization rates between the smallest particles and 500 nm particles were obvious (Figure 3)b. Quantification of the co-localization on a pixel basis using Metamorph software (Figure 3)c also showed significant differences between the co-localization of small (<100 nm) and larger particles after 4h, whereas after 24h only the co-localization rates between 20 nm and 40 nm versus 500 nm particles were found to be significantly different. There were no significant differences in the co-localization rate of the particles between the use of cathepsin B and of pH sensitive dye as lysosome marker.

### Lysosome function & integrity

To assess lysosomal morphology and function, dye-release induced by loss of lysosome membrane integrity, measurements of pH by fluorescent dye, a screening assay for lysosomal perturbation/dilation of the endosomal-lysosomal system in drug development and enzymatic assays for lysosome activity, were used. Chloroquine, a drug known to increase intralysosomal pH and to inhibit the activities of arylsulfatase B and cathepsin B activity [38,39], was used as positive control.

#### Changes in lysosomal membrane integrity, morphology and pH

Strong damage of lysosomal membranes leads to release of the fluorescent dye Lucifer yellow, whereas minor damage can lead to changes in the lysosomal pH identified by decrease in the staining with the indicator dye LysoSensor. As it may be difficult to detect small increases in lysosomal pH based on fluorescence, the switch of the Acridine Orange fluorescence from red in healthy lysosomes to green in lysosomes with increased pH was used in addition. Finally, also an assay used in the screening for lysomotropic effects (lysosomal perturbation assay) was used. This assay is based on the increase in the green fluorescence of NBZ-PZ relative to the red fluorescence of propidium iodide as indication for dilatation of the endosomal-lysosomal system. The effect of 20 μg/ml and 40 μg/ml CPS was investigated.

In the incubations with Lucifer Yellow the positive control (100 μM chloroquine) after 24h led to leakage of the dye into the cytoplasm resulting in a diffuse cytoplasmic staining instead of the punctate pattern in normal cells (Additional file 3: Figure S1, 20 μg/ml shown). The staining of cells exposed to 20 nm and 200 nm CPS for 24h was not different from not exposed cells. After 72h the signal was too low for evaluation.

Also the staining with LysoSensor did not show differences between 20 nm CPS, 200 nm CPS exposed cells (20 μg/ml and 40 μg/ml) and not exposed cells for 24h and 72h (data not shown).

**Figure 4:**
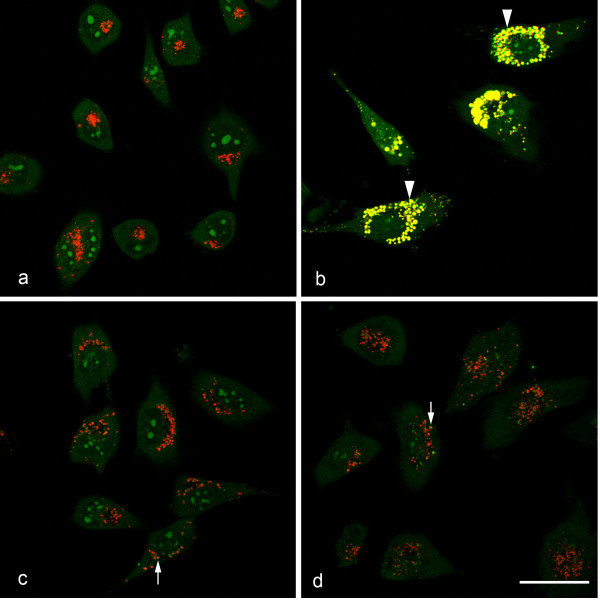
**EAhy 926 cells stained with acridine orange after treatment with DMEM + 2%FBS (a), 25 μM chloroquine (positive control, b), 20 μg/ml 20 nm CPS (c) and 20 μg/ml 200 nm CPS (d). DNA and RNA are stained green by acridine orange; healthy lysosomes stain only red.** Upon exposure to chloroquine (**b**) lysosomes increase in size and stain in green and red (yellow, arrow head) indicating increase in lysosomal pH and lysosomal swelling. Cells treated with CPS (**c**, **d**) show red lysosomes and only rarely double-stained yellow lysosomes (arrows). Scale bar: 10 μm

Morphology of acridine orange stained cells exposed to 25 μM chloroquine showed enlarged lysosomes with marked increase in green fluorescence as indication for lysosomal swelling (Figure 4p). Cells exposed to 20 μg/ml and 40 μg/ml 20 nm and 200 nm CPS as well as not particle exposed cells showed occasionally lysosomes with increased green staining but no enlargement of the organelles (Figure 4a, c, d, 20 μg/ml shown). To quantify these findings relative fluorescence in the red compared to the green channel was determined by fluorometry and a significant decrease in this ratio was seen for the positive control chloroquine (70.3 ± 2.2%). Neither in cells exposed to 20 μg/ml 20 nm CPS nor to those exposed to 20 μg/ml 200 nm CPS this ratio was significantly different from not exposed cells (91.37 ± 11.7% and 92.87 ± 13.2%, respectively).

**Figure 5:**
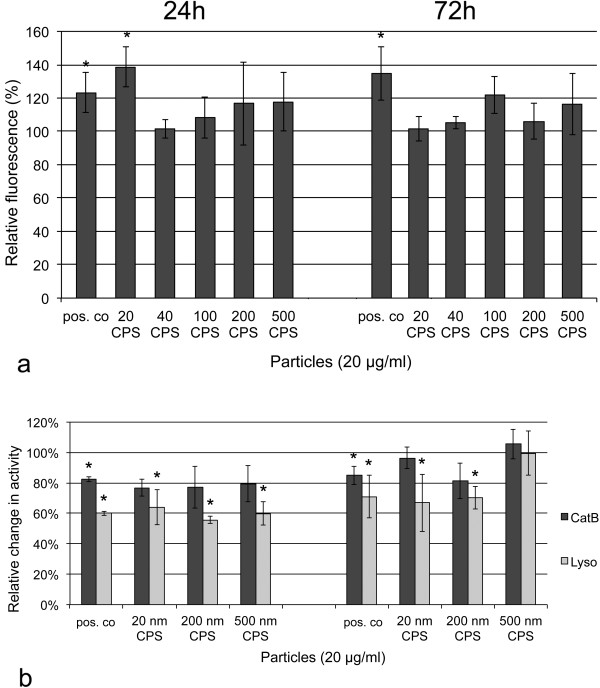
**Enlargement of the endosomal-lysosomal system and changes in lysosomal enzyme activities in EAhy926 cells after 24h and 72h of exposure to 20 μg/ml CPS of different sizes in DMEM + 2%FBS.** 25 μM chloroquine was used as positive control (pos. co). a: Lysosomal/cytotoxicity assay, where an increase in the green signal is interpreted as perturbation in lysosome function. The signal is normalized to that of un-treated cells as 100%. Significant effects (asterisk) are seen only after incubation for 24h with 20 nm CPS. b: Changes in the reactivity with CV-(RR)_2_ for cathepsin B (CatB) and with substrate for lysosomal sulfatases (Lyso). Arbitrary fluorescence data are normalized to the amount of LAMP-1 staining and the signal of un-treated cells is set as 100%. In general, decreases in lysosomal activities were more pronounced after 24h of exposure than after 72h of exposure and the effect on lysosomal sulfatases was higher than that on cathepsin B. After 24h of exposure, significant decreases in the activity of lysosomal sulfatase were seen for all particles. After 72h, such significant decreases were only seen upon exposure to 20 nm CPS and 200 nm CPS. Significant changes are marked by asterisks.

In the lysosomal perturbation assay, a significant increase in the green fluorescence was only seen for the exposure to 20 μg/ml and 40 μg/ml 20 nm CPS for 24h (Figure 5a, 20 μg/ml shown) and the positive control (25 μM chloroquine) at both time points. An increase in the amount of endosomal-lysosomal membranes was also seen when cells exposed for 24h to both concentrations of CPS were stained with anti-LAMP-1 antibody as marker for late endosomes and lysosomes. This increase to 135 ± 22% of the controls was not significant but was seen for all CPS-treated samples and at both concentrations of CPS (data not shown). After 72h of exposure to all particles, fluorescence in the green and red were similar to those of the untreated controls in the lysosomal perturbation assay (Figure 5a). Similarly, also the increase in the anti-LAMP-1 staining of CPS exposed cells was no more seen (104 ± 6% of the controls).

#### Changes in enzyme activity

The assessment of cathepsin B activity in homogenates did not produce reliable data and the *in-situ* detection was used instead. The *in-situ* detection of cathepsin B and lysosomal sulfatase activity is based on the diffusion of substrates into the lysosomes, where they are metabolized and result in a fluorescent product. The fluorescence was normalized to the intensity of the staining with anti-LAMP-1 antibody, as marker for the endosomal-lysosomal system since slight increases in LAMP-1 staining were seen upon exposure to CPS (see section lysosomal perturbation).

The decrease in lysosomal sulfatase activity was strongest after 24h of exposure to 20 μg/ml CPS (Figure 5b). 20 nm, 200 nm and 500 nm CPS decreased lysosomal sulfatase activity significantly; the decrease was most pronounced for 20 nm CPS. After 72h of exposure still significant decreases in lysosomal sulfatase activity were seen after exposure to 20 nm CPS and 200 nm CPS but not to 500 nm. Decrease of cathepsin B activity was not significant for these particles at both time points.

## Discussion

In this study the intracellular distribution of CPS, as models for non- biodegradable NPs, and their effect on lysosomes of the endothelial cell line EAhy926 was studied after 24h and 72h of exposure. These particles also served to identify assays, which could be used for the assessment of lysosomal effects of other NPs. 20 μg/ml and 40 μg/ml reacted similarly. In addition to dilatation of the endosomal-lysosomal system (lysosome/cytotoxicity dual staining kit), decreases in the activities of lysosomal enzymes (cathepsin B, lysosomal sulfatase) were seen. Overall, the observed effects were small and more pronounced at 24h than at 72h of incubation suggesting that prolonged contact does not cause lysosomal damage.

In this study the intracellular localization and effects on lysosomes of CPS were studied at 50 μg/ml as highest concentration in EAhy926 cells. These concentrations appear realistic for chronic effects upon repeated intravenous exposure and EAhy926 cells as endothelial cell lines are relevant target cells. In the cytostatic Abraxane® the drug load accounts for 10% of the total nanoparticle mass. Maximum serum concentrations for paclitaxel are 23 μg/ml [40], corresponding to nanoparticle concentrations of 230 μg/ml. Imaging agents based on gadolinium chelates may reach blood concentrations of 1 mg/ml (http://www.berlex.com/html/products/pi/Magnevist_PI.pdf), but blood levels for iron oxide NPs in Resovist® range around 100 μg/ml [41]. The given concentrations are peak levels and not typical for chronic exposure. On the other hand, cellular accumulation has been shown in animal experiments [5-9,42] and may maintain intracellular levels of NPs at a lower level over a prolonged period of time.

For the studies on lysosomes, we used serum-reduced cells to slow down proliferation and, thereby, prevent dilution of the intracellular nanoparticle concentration by cell division. Exposure to 0-1% FBS has been reported to decrease cell viability and induce apoptosis and necrosis in different cell lines [43-45]. In contrast to synthesis of RNA and DNA, protein synthesis was largely unaffected in L929 fibroblasts by serum deprivation but reduced in 3T3 fibroblasts [46,47]. To find out if serum deprivation displayed major effects on the EAhy926 cells used in this study, whole genome analysis was performed. As expected genes involved in pathways such as cell cycle, proliferation and apoptosis were changed but no effects on lysosomal genes were identified. The reduction of the serum content from 10% to 2%, therefore, appears to be suitable for the study of lysosomes.

As prerequisite for lysosomal accumulation, intracellular localization of fluorescent CPS was studied. Already after 4h of exposure, 20 nm CPS showed a high rate of co-localization to endosomes and lysosomes. Consistent with studies by Lai et al. [48] the co-localization rate of 40 nm CPS was higher than that of 20 nm CPS. Co-localization of > 40 nm CPS with lysosomes in our study increased with time and after 24h CPS also 500 nm CPS were localized in lysosomes. Although different uptake mechanisms have been reported, most NPs, like for instance mesoporous silica particles, silicon dioxide particles, gold particles and quantum dots predominantly were localized in lysosomes [49-52]. Size compared to surface charge appears to play only a minor role for lysosomal localization [53]. Nanoceria particles with positive and negative surface charge localized outside and inside lysosomes, respectively. Cytotoxicity data after 24h of exposure showed a much higher effect for lysosomal localization and the authors hypothesized that lysosomal localization is correlated to cytotoxicity on NPs in tumor cells. The authors do, however, not indicate if lysosomal changes are implicated in this tumor selective damage.

The occurrence of lysosomal damage without cell destruction is a well-known drug-induced lysosomal storage disorder termed lysosomal phospholipidosis. Several organic amines, namely chloroquine, amiodarone, perhexiline, aminoglycosides and chlorphentermine accumulate in the acidic environment of endosomes and lysosomes and cause the accumulation of membraneous material [54]. As positively charged molecules, they cause swelling and disruption of the lysosomes with subsequent cell death at high concentrations. Screening assays like the lysosomal/cytotoxicity dual staining kit or the Lyso-ID Red detection kit, both based on the combination of a membrane permeable dye with a marker for cell death or cell number, have been developed to identify drug-induced dilatation of the endosomal-lysosomal system due accumulation of lysosomal membranes [55,56]. Using the lysosomal/cytotoxicity dual staining kit, perturbation of lysosome function after exposure of cells to 20 nm CPS for 24h was identified. The interference with lysosome function of the 20 nm particles and absence of this effect of the 40 nm CPS may be caused by differences in the intracellular concentration of the particles that was about 8 times higher for 20 nm CPS than for 40 nm CPS. It is, therefore, likely that similar alterations also occur when the other CPS reach such high intracellular particle concentration. As CPS are not positively charged they are unlikely to disrupt lysosomes by neutralization of the acidic environment. Generation of oxidative stress, which has been demonstrated for fullerenes and for quantum dots [28,29] may also damage lysosomes. The size of quantum dots and fullerenes is roughly in the same order of magnitude as that of the 20 nm CPS, but it is not likely that CPS act mainly by generation of oxygen species. In the concentration range, where lysosomal perturbation by 20 nm CPS was recorded in this paper (20 μg/ml and 40 μg/ml), no significant increase in intracellular oxygen radicals was recorded. The lack of generation of oxygen radicals by CPS is consistent with other data [37,57]. Although no dramatic damage of lysosomal morphology was detected, we identified changes in enzymatic activities, especially of lysosomal sulfatase, upon exposure to CPS for 24h. These alterations were less pronounced at 72h of exposure. A more prominent decrease in enzyme activity at short incubation times than upon longer exposures is consistent with reported decreases in cathepsin B upon short exposure of macrophages to quartz D12 and to polystyrene microparticles [58,59]. Decreases in lysosomal sulfatase activities in this study were always greater than those in cathepsin B activity. This appears to be due to the specificity of the substrate used for the assessment. The substrate SulfGreen is metabolized by all lysosomal sulfatases (MarkerGene LysoLife product information: http://www.markergene.com/product_sheets/pis1377.pdf), whereas the specificity of the cathepsin B substrate for this protease is higher. Provided that CPS acted on all lysosomal enzymes in a similar manner, changes in SulfGreen fluorescence are expected to be greater than those in CV-(RR)_2_ (cathepsin B substrate) fluorescence.

In contrast to what was expected, damage by longer exposure to CPS was not higher than after shorter exposure. Polystyrene particles have a high potential for binding of proteins and form larger aggregates when in contact with protein-containing solutions [60,61]. It is, therefore, likely that the fraction of primary and potentially toxic particles rapidly declines inside the cell.

## Conclusions

For the CPS investigated in this study only little interference with lysosomal function was seen. Changes were more prominent upon short than upon longer exposure. It may be suspected that by aggregation inside lysosomes the reactivity of CPS is decreased and toxicity reduced. The panel of lysosomal assays, which was validated for CPS in this study, can be used as a tool to identify the action of other types of NPs on lysosomes.

## Methods

### Particles

Carboxyl polystyrene (CPS) latex beads (20, 40, 100, 200, 500 nm), and the respective fluorescently labelled carboxyl polystyrene latex beads (FluoSpheres®) were obtained from Invitrogen. The fluorescently labelled particles contain dyes integrated in the core, which has the advantage that fluorescent and non fluorescent particles have the same surface properties. CPS suspensions were put into an Elmasonic S40 water bath (ultrasonic frequency: 37 kHz, Elma, Singen) for 20 min prior to cell exposures and physicochemical characterization. To identify potential leakage of the dye from the FluoSpheres® after exposure for 24h-72h to cells at 37°C the supernatant of the cellular exposures was assessed for fluorescence. These measurements were performed only for the 500 nm spheres firstly because they contain the highest amount of fluorochrome and secondly because, according to the producer, it is not recommended to clean polystyrene spheres smaller than 300 nm by centrifugation. Supernatant containing the 500 nm FluoSpheres® were centrifuged at 20,000xg for 20 min in a Jouan 25 KRi centrifuge and fluorescence measured in a fluorescence plate reader (FLUOstar Optima, BMG Labortechnik) at 584 nm/612 nm. The fluorescence of the supernatant was 5 times higher than medium alone, this signal corresponds to 0.05% of the fluorescence of the stem solution. As the fluorochromes in the particles are subjected to quenching, whereas the leached fluorochromes are not, the real amount of leached dye is expected to be much lower.

### Physicochemical characterization of particles

Physicochemical characterization of the particles was performed by dynamic light scattering using a Malvern Zetasizer 3000 HS. Particles were diluted with respective medium to 200 μg/ml and sonicated. After equilibration of the sample solution to 25°C, size and zeta potential were measured at 633 nm and a detection angle of 90°. NNLS software was used for sample analysis.

### Cell culture

The human endothelial cell line EAhy926 (kind gift from Dr. C. J. Edgell) was cultured in DMEM, 10% fetal bovine serum (FBS), 2 mM L-glutamine and 1% penicillin/streptomycin. Cell numbers were determined by cell counting and analyser system (CASY® TT, Innovatis). The cells were exposed to particles in DMEM + 2 mM L-glutamine for acute cytotoxicity studies and in DMEM, 2% fetal bovine serum (FBS), 2 mM L-glutamine for assessment of lysosome function. All cells were cultured at 37° C in a humid 95% air/5% CO2 atmosphere. Cells were pre-cultured 24h in DMEM +10% FBS prior to the experiment. Subsequently, medium was removed and cells exposed to the particles suspended in DMEM + 2% FBS were cultured for up to 120h.

### RNA isolation

Total RNA was isolated using RNeasy Mini kit (Qiagen, Stanford, CA, USA) according to the manufacturer’s recommendations. The integrity of each RNA sample was evaluated using an Agilent 2100 Bioanalyzer (Agilent, Foster City, CA) and only RNAs with an RNA integrity number (RIN) above 9.5 were used for hybridizations.

### Hybridization of microarrays

100 ng of total RNA for each sample were processed using the Affymetrix GeneChip Whole Transcript (WT) Sense Target Labeling Assay according to the manufacturer’s instructions (Affymetrix). Double stranded cDNA was synthesized using a random hexamers tagged with a T7 promoter sequence. Using *in vitro* transcription, cRNA was generated from the double-stranded cDNA template using the Whole Transcript cDNA Synthesis and Amplification Kit (Affymetrix). cDNA was regenerated using a reverse transcription reaction randomly primed with a mix containing dUTP. After hydrolysis of the cRNA with RNase H, the sense strand of cDNA was purified using the Affymetrix sample cleanup module, fragmented by incubation with UDG (uracil DNA glycosylase) and APE 1 (apurinic/apyrimidic endonuclease 1), and terminally biotin-labeled with terminal deoxynucleotidyl transferase using the WT Terminal Labeling Kit (Affymetrix), following the manufacturer’s instructions. Biotinylated sense strands were fragmented and hybridized to Affymetrix Human GeneChip 1.0 ST arrays (Affymetrix) using the Hybridization Control and Hybridization Wash and Stain kits (Affymetrix). The hybridization cocktail was incubated overnight at 45°C while rotating in a hybridization oven. After 16 h of hybridization, arrays were washed and stained in an Affymetrix GeneChip fluidics station 450, according to the Affymetrix-recommended protocol. Arrays were scanned on an Affymetrix GeneChip scanner.

### Quantification of particle uptake

After 24h, 48h and 72h the incubation medium was removed, cells were washed three times with medium and removed from the plastic support by trypsin treatment (5 min with 0.25% trypsin/EDTA at 37°C). The action of trypsin was stopped with medium. One aliquot of the cell suspension was used for cell counting by CASY and another to measure fluorescence in a fluorescence plate reader (FLUOstar Optima, BMG Labortechnik) at 584 nm/612 nm.

To indicate particle uptake per cell the particle numbers in the stem solution used for the incubations were calculated according to the formula given by the producer (probes.invitrogen.com/media/pis/mp05000.pdf). The fluorescence signals of the stem solutions were measured by serial dilution. Cell suspensions instead of medium alone were used for the dilution to account for autofluorescence or quenching effects caused by the cells.

### Formazan bioreduction by MTS

CellTiter 96® AQueous Non-Radioactive Cell Proliferation Assay (Promega) was used according to the manufacturer’s instructions. In short, 20 μl of the combined MTS/PMS solution was added to 100 μl of each well. Plates were incubated for 2 hours at 37°C in the cell incubator. Absorbance was read at 490 nm on a plate reader (SPECTRA MAX plus 384, Molecular Devices).

### Evaluation of oxidative stress by oxidation of dichlorodihydrofluorescein

Cells were grown for 24h in cell culture plates and loaded with 10 μM 2,7-dichlorodihydrofluorescein diacetate (Invitrogen) in medium for 30 min at 37°C. Subsequently, cells were rinsed and cultured for 24h with 0-50 μg/ml CPS or 200 μM H_2_O_2_ as positive control. Fluorescence was read with 485 nm excitation and 520 nm emisssion at a FLUOstar Optima (BMG Labortechnik).

### Co-localization studies with lysosomes

For co-localization studies of particles and lysosomes, cells were loaded with 20, 40, 100, 200 nm and 500 nm FluoSpheres (25 μg/ml) for 4h. Subsequently, medium was replaced by fresh medium without particles and cells were cultured for a further 24h. Thereafter, cells were washed with PBS and stained for either pH (LysoSensor™ Green DND-189) or cathepsin B activity (CV-(RR)_2_), according to the protocol given below. Nuclei were stained by incubation with Hoechst33342 (1 μg/ml) for 15 min at RT. Images were taken with a LSM510 Meta confocal laser scanning microscope (Zeiss) with 405/BP420-480 for the blue channel, 488/BP 505–550 for the green channel (LysoSensor™, YG FluoSpheres®) and 543/LP 560 for the red channel (CV-(RR)_2_, red FluoSpheres®). The degree of co-localization was analysed in LSM images from YG FluoSpheres® and Ac-RR-AFC cathepsin substrate as well as from red FluoSpheres® and LysoSensor incubations. For analysis the COLOCAL plugin of Metamorph® 5.0 software (Visitron Systems) was used. Images were taken in the multitrack mode and images of the two channels were exported as .jgp files. Settings to suppress crosstalk between the channels had to be set for each particle separately because, as indicated by the producer, fluorescence intensity of the larger FluoSpheres®, due to higher fluorochrome content per particle, is much higher than that of 20 nm FluoSpheres®. Images were analysed without background subtraction with a threshold set at 100% for both channels. The area of overlap was measured and the data exported into Excel. In addition, co-localization was determined using the Metamorph plugin CORRPLOT. After putting the threshold at 100% for the red channel and for the green channel the correlation of both signals was analysed. 50 cells were analysed for each particle.

### Lysosomal membrane integrity

As positive control for lysosomal damage and interference with lysosome function chloroquine was used. After exposure for 24h to 20 nm and 200 nm CPS, to negative controls (medium) and to positive controls (25 μM chloroquine, Sigma-Aldrich) cells were washed and loaded with 1 μM Acridine Orange (Sigma-Aldrich) in PBS for 30 min at 37°C. After rinse in PBS cells fluorescence was determined at a FLUOstar Optima (BMG Labortechnik) with 485 nm/520 nm for the green and 584 nm/612 nm for the red channel. The ratio of the signal in the red to the green channel was determined.

Alternatively, Lucifer yellow was used for assessment of lysosomal integrity. Cells were loaded with 0.1 mg/ml Lucifer yellow dilithium salt (Sigma) in culture medium at 37°C for 16h prior to the exposure to the particles. Incubation with 100 μM chloroquine was used as positive control. Images were taken with a LSM510 Meta confocal laser scanning microscope (Zeiss) with ex 405 nm/em LP 505 nm.

### Assessment of lysosome function/pH

LysoSensor™ Green DND-189 (Invitrogen), an acidotropic probe, which accumulates in acidic compartments of cells as a result of protonization, was used at 1 μM for 5 min at 37°C, according to the user manual. Accumulation in an acidic environment results in a pH-dependent increase in fluorescence. Staining performed after preincubation for 4h with CPS of different sizes was compared to cells preincubated in medium only. Quantification was performed at a FLUOstar Optima (BMG Labortechnik) with 485 nm/520 nm and pictures were taken with a LSM510 Meta confocal laser scanning microscope (Zeiss) with ex 488/em BP 505–550.

Lysosome/Cytotoxicity Dual staining Kit (Cayman), as indicator for perturbation of lysosome function, was used as indicated by the producer. The membrane permeable 4-nitro-7-(1-piperazinyl)-2,1,3-benzoxadiazole (NBZ-PZ) reacts with carboxylic acids in the lysosome and fluorescence increases in an acid environment. Propidium iodide identifies cells with loss of membrane integrity. Chloroquine was used at a concentration of 25 μM as positive control. Data were acquired at a FLUOstar Optima (BMG Labortechnik) with 485 nm/520 nm for the green and 584 nm/612 nm for the red channel.

### Assessment of lysosome function/lysosomal enzymes

#### Cathepsin B activity

For detection in lysed cells the Cathepsin B Activity Assay Kit (PromoKine) with Ac-Arg-Arg labelled with amino-4-trifluoromethyl coumarine (Ac-RR-AFC) as substrate was used according to the instructions given in the User Manual. Fluorescence was measured at a FLUOstar Optima (BMG Labortechnik) using a 400 nm/505 nm filter set.

For the detection of cathepsin B activity *in-situ*, cells were rinsed in PBS and exposed to the CV-(RR)_2_ substrate of the BIOMOL CV- Cathepsin B Detection Kit (Eubio). The stem solution in DMSO was diluted 1:5000 with medium and added for 20 min at 37°C. After this incubation, cells were rinsed in PBS and fluorescence quantified by fluorometric reading on a FLUOstar Optima (584 nm/612 nm). Thereafter, cells were fixed in 4% formalin and processed for co-labelling with LAMP-1 antibody. In several experiments, fluorescence data of unfixed and fixed samples were compared to exclude an influence of the fixation on the result 25 µM chloroquine was used as positive control.

#### Lysosomal sulfatases

Activity *in situ* was assessed using the MarkerGene™ LysoLife™ Lysosomal Sulfatase Detection Kit (Eubio). After removal of the medium, the cells were washed once in PBS and incubated with the SulfGreen substrate (1.25 mM) in PBS for 4h at 37°C. After this incubation, cells were rinsed in PBS and fluorescence quantified by fluorometric reading on a FLUOstar Optima (485 nm/520 nm). Thereafter, cells were fixed in 4% formalin and processed for co-labelling with LAMP-1 antibody. In several experiments, fluorescence data of unfixed and fixed samples were compared to exclude an influence of the fixation on the result. 25 µM chloroquine was used as positive control.

#### Amount of lysosomes

For identification of changes in the endosomal-lysosomal system immunoreactivity against the major lysosomal membrane glycoprotein LAMP-1, located in the membrane of late endosomes and lysosomes [62], was used. Cells were fixed in 4% formalin for 10 min at RT, washed three times in PBS, blocked for 30 min in 1% goat serum and incubated with the anti LAMP-1 antibody (1:1000, rabbit, Abcam) for 1h at RT. For visualization of the antibody binding, different secondary antibodies were used. Alexa 488-labelled secondary antibody (1:200, goat anti-rabbit IgG, Invitrogen) in co-staining experiments with cathepsin B activity and DyLight 594-labelled secondary antibody (1:200, goat anti-rabbit IgG, ThermoScientific) in combination with lysosomal sulfatase activity was added for 30 min at RT. For microscopical images cells were counterstained with 1 μg/ml Hoechst33342 for 15 min at RT. Data were acquired at a FLUOstar Optima (BMG Labortechnik). The following filter settings were used for the fluorometric evaluation: 485 nm/520 nm (SulfGreen substrate, LAMP-1 green detection), 584 nm/612 nm (cathepsin B substrate CV-(RR)_2_, LAMP-1, red detection). For documentation of the staining, pictures were taken at a LSM510 Meta confocal laser scanning microscope (Zeiss) with 405/BP 420-480 for the blue channel, 488/BP 505–550 for the green channel (LysoSensor™, LAMP-1 detected with Alexa 488-labelled secondary antibody) and a 543/LP 560 for the red channel (CV-(RR)_2_, LAMP-1 detected with DyLight 594-labelled secondary antibody).

### Statistics

For Data Analysis of microarrays CEL files were imported into Partek Genomic Suite6.4 software (Partek Inc) and robust multi-chip average (RMA) normalized (including background correction, quantilequintile normalization across all arrays, median polished summarization based on log transformed expression values). For detection of differentially expressed genes analysis of variance (ANOVA) was performed and genes with FDR5% and a fold change of at least 2 were considered to be significantly de-regulated.

All other data are represented as means ± S.D from three to six experiments. Data were analyzed with one-way analysis of variance (ANOVA) followed by Tukey-HSD post hoc test for multiple comparisons (SPSS 19 software). Differences between two samples were analyzed by independent *t*-test and Levine's Test for Equality of Variances. Results with p-values of less than 0.05 were considered to be statistically significant.

## Competing interest

The authors disclose any financial competing interests.

## Authors’ contributions

EF: study design and writing of manuscript. CM, MA: experiments and data analysis. ER: particle characterization and draft of manuscript. EB: analysis of microarray data. TP: coordination and draft the manuscript. All authors read and approved the final manuscript.

## Supplementary Material

Additional file 1 **Table S1. Analysis of gene de-regulation in cells cultured in medium + 2% FBS compared to cells in medium + 10% FBS according to biological processes.** A. Up-regulated genes in 2% FBS compared to 10% FBS. B. Down-regulated genes in 2% FBS compared to 10% FBS. Click here for file

Additional file 2 Table S2. Click here for file

Additional file 3 **Figure S1. Staining of EAhy926 cells for lysosomal integrity using Lucifer yellow.** Controls show a punctate staining, whereas cells treated with chloroquine show a diffuse cytoplasmic staining. The staining pattern of cells treated with 20 μg/ml 20 nm and 200 nm carboxyl polystyrene particles (CPS) is similar to that of untreated controls. scale bar: 20μm. Figure 5s: Confocal image of LysoSensor (green) and cathepsin B substrate CV-(RR)_2_ (red) double-stained EAhy926 cells. Co-localization of both staining is seen in yellow. In general more structures with acid content stained with LysoSensor than those with cathepsin B activity are seen. Arrows mark organelles with high cathepsin B activity and arrowheads indicate acidic structures with low cathepsin B activity. Scale bar: 10μm. Click here for file
