# Supplementary material for: Action of polystyrene nanoparticles of different sizes on lysosomal function and integrity
Source: Part Fibre Toxicol. 2012 Jul 12;9:26. doi: 10.1186/1743-8977-9-26 (PMC3425083; doi:10.1186/1743-8977-9-26)
Supplement: Additional file 1 — Table S1. Analysis of gene de-regulation in cells cultured in medium + 2% FBS compared to cells in medium + 10% FBS according to biological processes. A. Up-regulated genes in 2% FBS compared to 10% FBS. B. Down-regulated genes in 2% FBS compared to 10% FBS. [file 1743-8977-9-26-S1.doc]

Table Is: Analysis of gene de-regulation in cells cultured in medium + 2% FBS compared to cells in medium + 10% FBS according to biological processes.

A. Up-regulated genes in 2% FBS compared to 10% FBS

| Biological Process | Genes on array | changed in samples | over/under | p-value |
| --- | --- | --- | --- | --- |
| metabolic process | 8267 | 287 | + | 5,39E-08 |
| primary metabolic process | 7950 | 274 | + | 3,60E-07 |
| cellular process | 6258 | 184 | + | 1,12E-01 |
| Unclassified | 6681 | 170 | - | 1,51E-01 |
| protein metabolic process | 3240 | 142 | + | 3,01E-09 |
| cell communication | 4365 | 137 | + | 3,48E-02 |
| signal transduction | 4191 | 133 | + | 2,78E-02 |
| immune system process | 2628 | 126 | + | 1,43E-10 |
| nucleobase, nucleoside, nucleotide and nucleic acid metabolic process | 3825 | 118 | + | 7,39E-02 |
| transport | 2857 | 103 | + | 1,72E-03 |
| developmental process | 3001 | 100 | + | 1,82E-02 |
| response to stimulus | 1798 | 99 | + | 1,60E-11 |
| system development | 2031 | 70 | + | 2,46E-02 |
| cell surface receptor linked signal transduction | 2235 | 68 | + | 1,81E-01 |
| mesoderm development | 1528 | 60 | + | 2,99E-03 |
| intracellular protein transport | 1646 | 58 | + | 2,71E-02 |
| protein transport | 1646 | 58 | + | 2,71E-02 |
| cell cycle | 1840 | 57 | + | 1,70E-01 |
| cell-cell signaling | 1331 | 55 | + | 1,53E-03 |
| intracellular signaling cascade | 1568 | 54 | + | 4,59E-02 |
| system process | 2216 | 53 | - | 1,76E-01 |
| neurological system process | 1954 | 49 | - | 3,02E-01 |
| ectoderm development | 1426 | 45 | + | 1,71E-01 |
| immune response | 756 | 45 | + | 1,24E-06 |
| vesicle-mediated transport | 1160 | 41 | + | 5,51E-02 |
| nervous system development | 1258 | 40 | + | 1,76E-01 |
| carbohydrate metabolic process | 952 | 38 | + | 1,32E-02 |
| apoptosis | 966 | 37 | + | 2,50E-02 |
| cellular component organization | 1443 | 35 | - | 2,70E-01 |
| cell adhesion | 1333 | 35 | - | 4,56E-01 |
| response to stress | 500 | 33 | + | 4,29E-06 |
| cellular defense response | 457 | 28 | + | 8,06E-05 |
| lipid metabolic process | 1119 | 27 | - | 2,97E-01 |
| ion transport | 739 | 27 | + | 7,80E-02 |
| cell motion | 964 | 25 | - | 4,53E-01 |
| synaptic transmission | 594 | 24 | + | 3,81E-02 |
| anatomical structure morphogenesis | 1121 | 23 | - | 9,17E-02 |
| cellular component morphogenesis | 1121 | 23 | - | 9,17E-02 |
| cellular amino acid and derivative metabolic process | 367 | 23 | + | 2,50E-04 |
| reproduction | 1003 | 22 | - | 1,73E-01 |
| cell-cell adhesion | 799 | 22 | + | 5,08E-01 |
| cation transport | 621 | 21 | + | 1,85E-01 |
| gamete generation | 920 | 20 | - | 1,77E-01 |

B. Down-regulated genes in 2% FBS compared to 10% FBS

| Biological Process | Genes on array | changed in samples | over/under | P-value |
| --- | --- | --- | --- | --- |
| cellular process | 6258 | 226 | + | 2,44E-18 |
| metabolic process | 8267 | 201 | + | 3,92E-02 |
| primary metabolic process | 7950 | 195 | + | 3,10E-02 |
| Unclassified | 6681 | 137 | - | 1,61E-01 |
| cell communication | 4365 | 122 | + | 2,24E-03 |
| signal transduction | 4191 | 117 | + | 2,96E-03 |
| nucleobase, nucleoside, nucleotide and nucleic acid metabolic process | 3825 | 92 | + | 1,92E-01 |
| developmental process | 3001 | 86 | + | 6,17E-03 |
| cell cycle | 1840 | 86 | + | 2,55E-11 |
| transport | 2857 | 85 | + | 2,38E-03 |
| protein metabolic process | 3240 | 84 | + | 6,17E-02 |
| cellular component organization | 1443 | 80 | + | 2,99E-14 |
| immune system process | 2628 | 63 | + | 2,57E-01 |
| system process | 2216 | 63 | + | 2,20E-02 |
| protein transport | 1646 | 56 | + | 8,70E-04 |
| intracellular protein transport | 1646 | 56 | + | 8,70E-04 |
| neurological system process | 1954 | 55 | + | 3,69E-02 |
| cellular component morphogenesis | 1121 | 54 | + | 8,36E-08 |
| anatomical structure morphogenesis | 1121 | 54 | + | 8,36E-08 |
| cell adhesion | 1333 | 49 | + | 3,59E-04 |
| intracellular signaling cascade | 1568 | 45 | + | 4,33E-02 |
| vesicle-mediated transport | 1160 | 44 | + | 3,82E-04 |
| mitosis | 635 | 44 | + | 4,21E-11 |
| system development | 2031 | 43 | - | 4,28E-01 |
| response to stimulus | 1798 | 41 | + | 4,34E-01 |
| cell surface receptor linked signal transduction | 2235 | 38 | - | 4,78E-02 |
| mesoderm development | 1528 | 38 | + | 2,43E-01 |
| lipid metabolic process | 1119 | 32 | + | 8,24E-02 |
| cell-cell signaling | 1331 | 27 | - | 3,71E-01 |
| apoptosis | 966 | 27 | + | 1,26E-01 |
| ectoderm development | 1426 | 26 | - | 1,81E-01 |
| cell motion | 964 | 26 | + | 1,71E-01 |
| organelle organization | 328 | 26 | + | 3,58E-08 |
| cell-cell adhesion | 799 | 25 | + | 5,27E-02 |
| establishment or maintenance of chromatin architecture | 302 | 25 | + | 2,91E-08 |
| chromosome segregation | 206 | 25 | + | 1,40E-11 |
| carbohydrate metabolic process | 952 | 22 | + | 4,41E-01 |
| sensory perception | 708 | 22 | + | 7,00E-02 |
