# Supplementary material for: Action of polystyrene nanoparticles of different sizes on lysosomal function and integrity
Source: Part Fibre Toxicol. 2012 Jul 12;9:26. doi: 10.1186/1743-8977-9-26 (PMC3425083; doi:10.1186/1743-8977-9-26)
Supplement: Additional file 3 — Figure S1. Staining of EAhy926 cells for lysosomal integrity using Lucifer yellow. Controls show a punctate staining, whereas cells treated with chloroquine show a diffuse cytoplasmic staining. The staining pattern of cells treated with 20 μg/ml 20 nm and 200 nm carboxyl polystyrene particles (CPS) is similar to that of untreated controls. scale bar: 20μm. Figure 5s: Confocal image of LysoSensor (green) and cathepsin B substrate CV-(RR)2 (red) double-stained EAhy926 cells. Co-localization of both staining is seen in yellow. In general more structures with acid content stained with LysoSensor than those with cathepsin B activity are seen. Arrows mark organelles with high cathepsin B activity and arrowheads indicate acidic structures with low cathepsin B activity. Scale bar: 10μm. [file 1743-8977-9-26-S3.doc]

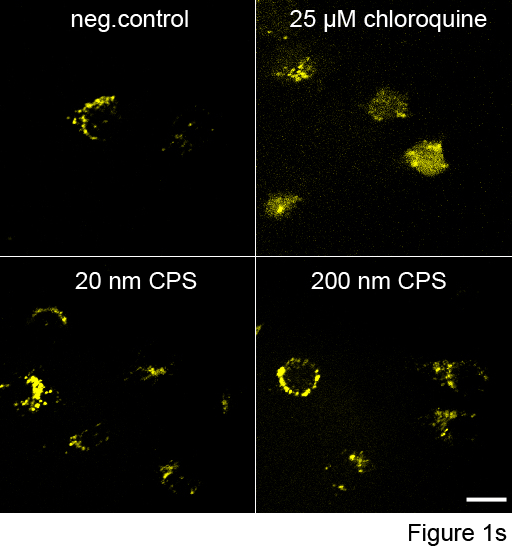


Fig. 1s: Staining of EAhy926 cells for lysosomal integrity using Lucifer yellow. Controls show a punctate staining, whereas cells treated with chloroquine show a diffuse cytoplasmic staining. The staining pattern of cells treated with 20 µg/ml 20 nm and 200 nm carboxyl polystyrene particles (CPS) is similar to that of untreated controls. scale bar: 20µm


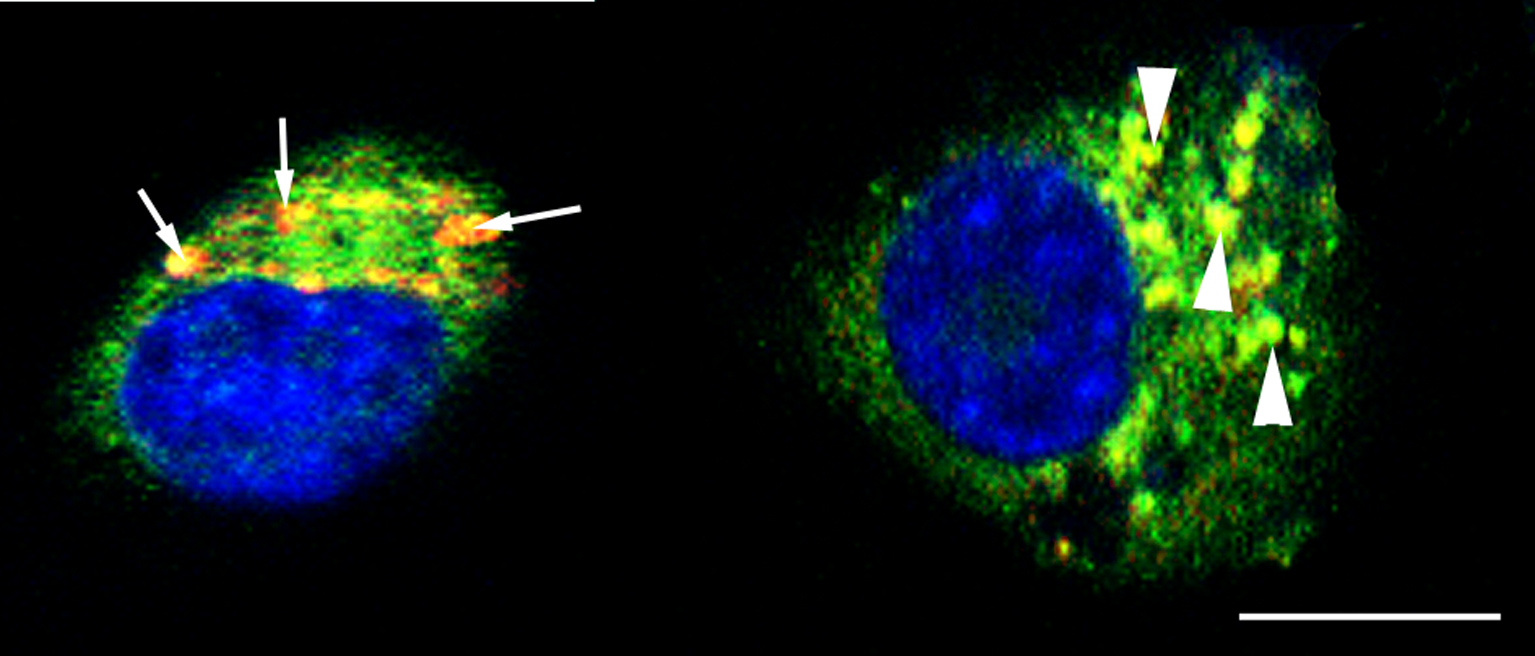


Fig. 5s: Confocal image of LysoSensor (green) and cathepsin B substrate CV-(RR)_2_ (red) double-stained EAhy926 cells. Co-localization of both staining is seen in yellow. In general more structures with acid content stained with LysoSensor than those with cathepsin B activity are seen. Arrows mark organelles with high cathepsin B activity and arrowheads indicate acidic structures with low cathepsin B activity. Scale bar: 10µm
